# From genes to networks: in systematic points of view

**DOI:** 10.1186/1752-0509-5-S3-I1

**Published:** 2011-12-23

**Authors:** Ke Zhang, Yunlong Liu, Jack Y Yang, Hamid R Arabnia, Andrzej Niemierko, Arif Ghafoor, Weizhong Li, Youping Deng

**Affiliations:** 1 Department of Pathology, Bioinformatics Core, School of Medicine and Health Sciences, University of North Dakota, Grand Forks, ND 58201, USA; 2 Center for Computational Biology and Bioinformatics, Indiana University School of Medicine, Indianapolis, IN, USA; 3 Department of Medical and Molecular Genetics, Indiana University School of Medicine, Indianapolis, IN, USA; 4 Center for Medical Genomics, Indiana University School of Medicine, Indianapolis, IN, USA; 5 Center for Research in Biological Systems, University of California at San Diego, La Jolla, California 92093-0043, USA; 6 Department of Radiation Oncology, Massachusetts General Hospital and Harvard Medical School, Harvard University, Boston, Massachusetts 02114, USA; 7 Department of Computer Science, University of Georgia, Athens, Georgia 30602-7404, USA; 8 Purdue School of Electrical and Computer Engineering, Purdue University, West Lafayette, IN 47907, USA; 9 Department of Internal Medicine, Rush University Medical Center, Chicago, IL 60612, USA; 10 Rush University Cancer Center, Rush University Medical Center, Chicago, IL 60612, USA; 11 Department of Biochemistry, Rush University Medical Center, Chicago, IL 60612, USA

## Abstract

We present a report of the BIOCOMP'10 - The 2010 International Conference on Bioinformatics & Computational Biology and other related work in the area of systems biology.

## 

This supplement to BMC Systems Biology was proposed to launch during the BIOCOMP'10 in Las Vegas, Nevada on July 18-21, 2010. In the BIOCOMP'10, many of the presentations were focused on global gene or protein expression and interaction using advanced computational and statistical methods. We accept submissions both from the presenters at the congress and from non-presenters. After rigorous peer review, we selected 14 papers that investigated the forefront systems biology field and that showed the state of the art technology for publication in this supplement.

There is an array of manuscripts focused on analysis of gene regulatory pathway and gene network. The first step of pathway or network analysis is to identify the differentially expressed gene from global gene expression profiles. RNA microarray has been widely used to measure the global gene expression. In the last few years, the fast development of next-generation sequencing technology has made RNA-Seq an alternative approach to obtain more accurate and comprehensive gene expression profiles. Nonetheless, bioinformatics tools are underdeveloped to cope with the fast increase of RNA-Seq data. One manuscript written by Chen and his colleagues [1] addressed the question of statistical test for differentially expressed genes from RNA-Seq experiment. They proposed a Wald-log test that was targeted to detect the difference between the low RNA expression levels when the traditional methods have little statistical power.

Time course microarray experiment has become an important design to understand biological dynamics and the underlying gene regulatory mechanism. Several manuscripts were devoted to derive gene pathway and network from time series gene expression data. Jin and her colleagues [2] developed a novel method by incorporating differential equations with a Bayesian method that can effectively model linear and nonlinear dynamic changes in transcriptional levels. Due to the computational inefficiency of Bayesian network methods, Zhang and his colleagues [3] chose to use State Space Model (SSM) to construct the gene regulatory networks for time course microarray data. This SSM based method is computationally faster than Dynamic Bayesian Network, thus it is capable to deal with larger number of genes. A striking application of gene network analysis is to investigate the dynamic changes of transcription factor levels during pollen development. A manuscript by multiple authors including Tieliu Shi [4] reported a meta-analysis of gene expression data for *Arabidopsis thaliana *pollen development. They constructed a network consisting of 19 transcription factors and 101 target genes using Network Component Analysis.

Disease diagnosis and prediction using machine learning methods such as genomic signature identification and disease sample classification are the emerging bioinformatics areas drawing high attention currently. In order to obtain reliable gene expression signatures for breast cancer, Wang and his colleagues [5] integrated various breast cancer signatures using graph centrality in a context-constraint protein interaction network. The resulting gene signatures were validated in microarray data and strong association with clinical outcomes were found. Pan and his colleagues [6] used multiple methods to detect important network motifs for gene regulatory networks. Their framework of network analysis has potential applications in disease signature studies. One of the examples is presented by Zhao and his colleagues [7], who conducted network analysis to identify genomic signatures for major depressive disorder (MDD). They pooled multiple source of information including association, linkage, gene expression, regulatory pathway, and literature search and found two unique network motifs that were enriched by MDD. Microarray data is known to have high dimensional features and high level of noises that present hurdles for efficient computation and accurate disease prediction. Land *et al*. [8] applied Kernelized Partial Least Squares to address this question by feature selection. The lung adenocarcinoma samples classified by their proposed method were shown a good association to clinical survival data.

Not all authors of network analysis devoted themselves on genomics data. Protein data is another focus to study gene interaction and system behaviour. A research team including Xu and Shi [9] proposed a novel way to classify types of viruses by looking into the virus-host protein-protein network. This method can reveal important protein-protein interactions as well. Chen and his colleagues [10] performed an integrative analysis for proteomics data to investigate the protein level changes during rat bone cells stimulation in response to *normal loading *and *fatigue loading*. Protein-protein interaction network was built based on the differentially expressed proteins.

This supplement also includes manuscripts using various other data types for systematic studies. Chun and his colleagues [11] targeted to improve mammography diagnosis for breast cancer. They proposed to use watershed transform for automatic mass segmentation and to use various noise reduction techniques to refine the segmentation. Xie *et al*. [12] provided a unique view the human tissue differentiation by combining global DNA methylation and RNA-Seq data. They had an amazing founding that DNA methylation and gene expression are universally correlated and their joint pattern can be used to investigate the genetic mechanism of tissue differentiation. There is work to extend network analysis to large scales, such as population and evolution. Marinescu and her colleagues [13] leveraged social networks to ecology for modelling the propagation of the influenza virus. This work helps to understand how infectious agents disseminate throughout a population. The research team consisting of Lin, Zhang and Xu [14] proposed a method to classify bifurcating branches of a phylogenetic tree into a set of polytomies that reflect more accurate evolutionary relationship.

In summary, this supplement to BMC Systems Biology presents a number of high quality articles in a wide range of areas in systems biology. These articles provide up-to-date information of research development in bioinformatics fields in terms of systems biology. We hope you find this issue as useful and as interesting as we do.
